# Maintenance Fluid Therapy with Saline, Dextrose-Supplemented Saline or Lactated Ringer in Childhood: Short-Term Metabolic Effects

**DOI:** 10.3390/nu12051449

**Published:** 2020-05-17

**Authors:** Alessandra Ricciuti, Gregorio P. Milani, Silvia Tarantino, Roberta Ghilardi, Sebastiano A.G. Lava, Marco Alberzoni, Mario G. Bianchetti, Carlo Agostoni

**Affiliations:** 1 Pediatric Unit, Fondazione IRCCS Ca’ Granda Ospedale Maggiore Policlinico, 20122 Milan, Italy; alessandra.ricciuti@studenti.unimi.it (A.R.); silvia.tarantino@policlinico.mi.it (S.T.); robighILA@yahoo.it (R.G.); carlo.agostoni@unimi.it (C.A.); 2 Department of Clinical Sciences and Community Health, Università degli Studi di Milano, 20122 Milan, Italy; alberzoni.marco@gmail.com; 3 Italian Society for Pediatric Gastroenterology Hepatology and Nutrition (SIGENP), 20126 Milan, Italy; 4 Pediatric Cardiology Unit, Department of Pediatrics, Centre Hospitalier Universitaire Vaudois, and University of Lausanne, 1010 Lausanne, Switzerland; webmaster@sebastianolava.ch; 5 Università della Svizzera Italiana, 6900 Lugano, Switzerland; mario.bianchetti@usi.ch

**Keywords:** metabolic acidosis, ketosis, fasting, isotonic solutions, fluids, hydration

## Abstract

Maintenance with isotonic fluids is recommended in children with gastroenteritis and failure of oral rehydration therapy. However, little is known on the short-term effects of the commonly prescribed intravenous solutions on metabolic balance in children. The aim of this study is to report on our experience with normal saline, dextrose-supplemented saline and lactated Ringer solution. Methods: A retrospective analysis from the charts of all previously apparently healthy children with acute gastroenteritis, mild to moderate dehydration and failure of oral rehydration, evaluated between January 2016 and December 2019 at our institution, was performed. Subjects prescribed the above-mentioned maintenance intravenous fluids and with blood testing immediately before starting fluid therapy and 4–6 h later, were eligible. The changes in bicarbonate, ionized sodium, potassium, chloride, anion gap and glucose were investigated. Kruskal–Wallis test with the post-hoc Dunn’s comparison and the Fisher exact test were applied. Results: A total of 134 out of 732 children affected by acute gastroenteritis were included (56 patients were prescribed normal saline, 48 dextrose-supplemented normal saline and 30 lactated Ringer solution). The effect of the three solutions on sodium and potassium was similar. As compared to non-supplemented normal saline (+0.4 (−1.9 – +2.2) mmol/L), dextrose-supplemented normal saline (+1.5 (+0.1 – +4.2) mmol/L) and lactated Ringer (+2.6 (+0.4 – +4.1) mmol/L) solution had a positive effect on plasma bicarbonate. Finally, the influence of dextrose-supplemented saline on blood glucose was different (+1.1 (+0.3 – +2.2) mmol/L) compared to that observed in cases hydrated with non-supplemented saline (−0.4 (−1.2 – +0.3) mmol/L) or lactated Ringer solution (−0.4 (−1.2 – +0.1) mmol/L). Conclusions: This study points out that maintenance intravenous therapies using normal saline, dextrose-supplemented saline or lactated Ringer solution have different effects on metabolic balance. A personalized fluid therapy that takes into account the clinical and biochemical variables is advised.

## 1. Introduction

It has been more than 10 years since the maintenance of intravenous fluid therapy with isotonic normal saline was advised for pediatric inpatients based on the increased risk of hyponatremia with the use of “traditional” hypotonic solutions [1]. More recently, normal saline supplemented with dextrose has been proposed to prevent the development of hypoglycemia. Finally, lactated Ringer solution, an isotonic buffered crystalloid, has also been suggested to prevent hyperchloremic acidosis [1,2,3].

The short-term effect of normal saline, dextrose-supplemented saline and lactated Ringer solutions on sodium, potassium, chloride, acid-base and glucose balance has so far been only marginally addressed in childhood. Unsurprisingly, therefore, currently available guidelines do not state the preferred isotonic solution for intravenous fluid maintenance [4].

This observational retrospective study aims to investigate the short-term metabolic changes secondary to normal saline, dextrose-supplemented saline and lactated Ringer’s solution maintenance therapy in children with mild to moderate acute gastroenteritis and failure of oral rehydration therapy.

## 2. Subjects and Methods

A semi-structured hydration management is applied for children 4 weeks to 17 years of age with acute onset of three or more episodes of loose stools per day (or a number of bowel movements exceeding the customary number of daily bowel movements by two or more) with or without vomiting [5,6], who are presented to the Pediatric Emergency Department, Ca’ Granda Ospedale Maggiore Policlinico, Milan, Italy. Briefly, intravenous fluid repair is prescribed for cases with severe dehydration while children with moderate, minimal or no dehydration are encouraged to continue their usual diet plus drink adequate fluids. Finally, in cases with mild to moderate dehydration and failure of oral rehydration therapy, venous blood is sampled anaerobically with minimal stasis for the determination of electrolyte, acid-base and glucose balance, and maintenance intravenous fluid therapy is prescribed at a rate of 70 mL/m^2^ body surface area per hour.

The following three crystalloids are available: normal saline (sodium 154 mmol/L, chloride 154 mmol/L), normal saline supplemented with 5% dextrose (sodium 154 mmol/L, chloride 154 mmol/L, glucose 50 g/L) and lactated Ringer solution (sodium 130 mmol/L, chloride 109 mmol/L, lactate 28 mmol/L, potassium 4.0 mmol/L, calcium 1.5 mmol/L). It is at the physician’s own discretion whether blood testing for sodium, potassium, chloride, bicarbonate and glucose is repeated 4–6 h later.

The data for the present retrospective analysis were anonymized and extracted from the charts of all children with acute gastroenteritis evaluated between January 2016 and December 2019, who fulfilled the following inclusion criteria: 1. they were previously an apparently healthy individual; 2. they experienced diagnosis of acute gastroenteritis with mild to moderate dehydration and failure of oral rehydration therapy; 3. intravenous fluid therapy was maintained at a constant rate of 70 mL/m^2^, and 4. blood testing was performed as indicated above immediately before starting fluid therapy and 4–6 h later.

Ionized sodium, chloride, potassium, pH and carbon dioxide pressure (direct potentiometry), glucose and L-lactate (amperometry) were determined in whole blood in a GEM Premier TM 4000 analyzer [7]. Furthermore, plasma bicarbonate was estimated from pH and carbon dioxide pressure and blood anion gap was calculated by subtracting the concentrations of chloride and bicarbonate from that of sodium [7]. Blood was again sampled if potassium was ≥5.5 mmol/L or lactate ≥2.5 mmol/L and the lowest value was employed [7].

Continuous data are given either as median and interquartile range or as box-and-whisker diagram and categorical data as frequency. The two-tailed Kruskal–Wallis test with the post-hoc Dunn’s comparison was used to compare continuous variables and the two-tailed Fisher exact test to compare categorical variables among groups. Statistical significance was assigned at *P* < 0.05. The statistical analysis was performed using the R software, version 3.5.3 (2019-03-11).

## 3. Results

### 3.1. Baseline Data

Between January 2016 and December 2019, 134 out of 732 children affected by acute gastroenteritis fulfilled all inclusion criteria in this analysis (Table 1). Females (2.5 (1.6–5.8) years) and males (2.3 (1.1–8.9) years) did not significantly differ with respect to age. A total of 56 patients were prescribed normal saline, 48 dextrose-supplemented normal saline and 30 lactated Ringer solution. Some heterogeneity was observed in the baseline characteristics of the three groups. As compared to the remaining two groups, children assigned to receive dextrose-supplemented saline were predominantly female, while those prescribed lactated Ringer solution were younger. Female and male patients did not significantly differ with respect to age in the group prescribed normal saline (4.9 (2.5–6.2) versus 2.9 (1.1–12) years), in the group prescribed dextrose-supplemented saline (2.2 (1.6–2.8) versus 2.6 (1.2–6.1) years) and in the group prescribed lactated Ringer (1.7 (1.0–3.4) versus 1.9 (1.1–3.5) years). Baseline blood glucose was lower in patients prescribed dextrose-supplemented saline than in those prescribed non-supplemented saline or lactated Ringer solution. At baseline, whole blood chloride was higher and plasma bicarbonate was lower in children prescribed lactated Ringer solution as compared to those prescribed normal saline (with or without dextrose supplementation). Finally, baseline blood anion gap was higher in patients prescribed dextrose-supplemented saline as compared to those prescribed non-supplemented saline. Baseline circulating L-lactate was always <2.5 mmol/L.

### 3.2. Effects of Maintenance Fluid Therapy

Sodium, potassium, chloride, bicarbonate and anion gap were similar and L-lactate was always <2.5 mmol/L in the three study groups 4–6 h after intravenous maintenance therapy. In contrast, blood glucose was significantly higher in the group of patients hydrated with dextrose-supplemented saline as compared with the two remaining groups (Table 1).

**Figure 1:**
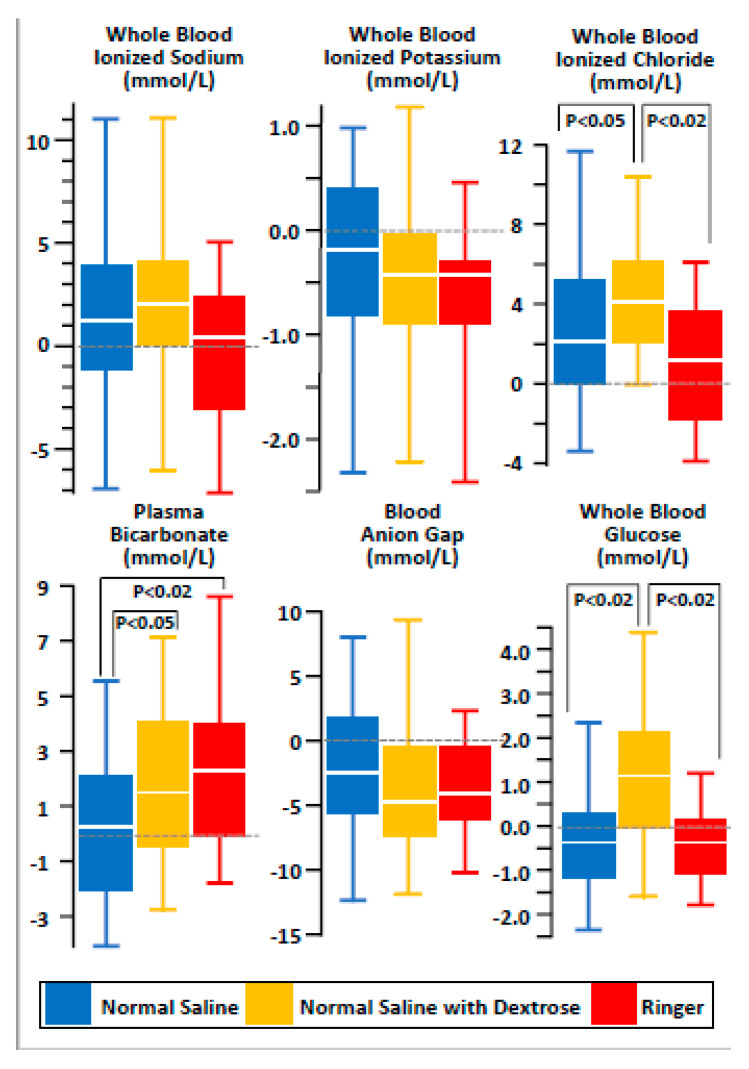
Changes in ionized sodium, potassium, chloride, bicarbonate, anion gap and glucose after short-term maintenance intravenous fluid therapy using normal saline (N = 56), dextrose-supplemented saline (N = 48) or lactated Ringer’s (N = 30) solution in children with mild to moderate acute gastroenteritis and failure of oral rehydration therapy. The results are given as “box-and-whisker diagram”: bottom and top of box represent the 25th and the 75th centile, respectively, middle of box the 50th centile (the median), ends of whiskers the 5th and the 95th centile, respectively.

The effect of normal saline (without or with dextrose supplementation) and lactated Ringer’s solution on sodium and potassium was similar (Figure 1). As compared to non-supplemented normal saline (+0.4 (−1.9 – +2.2) mmol/L), dextrose-supplemented normal saline (+1.5 (+0.1 – +4.2) mmol/L) and lactated Ringer’s (+2.6 (+0.4 – +4.1) mmol/L) solution had a positive effect on plasma bicarbonate. Finally, the influence of dextrose-supplemented normal saline on blood glucose was different (+1.1 (+0.3 – +2.2) mmol/L) compared to non-supplemented normal saline (−0.4 (−1.2 – +0.3) mmol/L) or lactated Ringer’s solution( −0.4 (−1.2 – +0.1) mmol/L).

## 4. Discussion

This retrospective analysis of our experience with maintenance intravenous fluid therapy for 4–6 h in children with mild to moderate acute gastroenteritis and failure of oral rehydration may be summarized as follows: (a) contrary to non-supplemented normal saline and lactated Ringer’s solution, the administration of dextrose-supplemented normal saline was followed by an increase in blood glucose; (b) lactated Ringer’s solution and dextrose-supplemented normal saline were superior to non-supplemented normal saline in increasing plasma bicarbonate (on the other hand, hydration with normal saline was not followed by hyperchloremic acidosis); (c) the effect of lactated Ringer’s solution and normal saline without and with dextrose supplementation on circulating potassium and sodium was similar.

Ionized sodium level was low, i.e., ≤134 mmol/L in the majority of gastroenteritis cases included in this report. Until the eighties, hyponatremia was unusual in gastroenteritis [7]. In the following decades, the frequency of hyponatremia increased [7]. The renaissance of breastfeeding, low-salt formulas and the currently recommended early and fast reintroduction of mostly hypotonic fluids in gastroenteritis likely explain this shift [7].

The effects on blood glucose level observed after dextrose-supplemented saline, non-supplemented saline or lactated Ringer’s solution are not surprising. While the effect of lactated Ringer’s solution on circulating bicarbonate may be anticipated, the effect of dextrose-supplemented saline seems unexpected at first sight and deserves some speculation. Fasting ketosis and acidosis are an increasingly recognized derangement in infants and younger children with gastroenteritis and mild to moderate dehydration [8,9]. The baseline laboratory features noted in patients assigned to be hydrated with dextrose-supplemented saline, which included rather low glucose and bicarbonate, absent hyperlactatemia and high anion gap, support this notion. Hence, we hypothesize that the increase in bicarbonate and glucose observed after the administration of dextrose results from a partial correction of ketosis [8,9].

Lactated Ringer’s solution is customarily contraindicated in hyperkalemia because it contains potassium [2,8]. Our experience, consistent with the very recent literature, does not support this view [2,8]. Two explanations underlie this observation: (a) the potassium contained in lactated Ringer, which is only 4.0 mmol/L, promptly dissolves in the extracellular space; (b) contrary to normal saline, which may cause “dilutional” hyperchloremic metabolic acidosis, lactated Ringer’s solution does not mobilize potassium from the intracellular space [2,8].

The sodium concentration is lower by approximately 15% (130 versus 154 mmol/L) with Ringer’s solution as compared with normal saline [2,3,10]. Nonetheless, in this short-term experience, the effect of both solutions on circulating sodium was similar. Normal saline did not elevate circulating sodium because this solution, which is often improperly described to be slightly hypertonic and subsequently capable of triggering an increase in sodium concentration, is isotonic to plasma and not associated with hypernatremia, as recently discussed [11]. On the other hand, currently available balanced solutions including lactated Ringer are slightly hypotonic to plasma (sodium approximately 120 mmol/L) and might be associated with hyponatremia [11].

Our study presents both limitations and strengths. As a consequence of the retrospective design, the three groups were rather heterogeneous with respect to gender, age and baseline glucose, anion gap and bicarbonate levels. Furthermore, we did not collect information on renal function such as urine output and creatinine levels in children with mild to moderate acute gastroenteritis. Moreover, the metabolic impact at 4–6 h may be uneasily extrapolated at 18–24 h. Finally, urinary ketones were not tested. On the other hand, as a major strength, the management of children with mild to moderate acute gastroenteritis is well defined and structured at our institution. This attitude made it possible to detect even subtle but still significant changes in salt, acid-base and glucose balance 4–6 h after starting maintenance fluid therapy with three different isotonic solutions. Moreover, sodium was determined by direct potentiometry, as is nowadays recommended [12,13].

## 5. Conclusions

These preliminary data point out that maintenance intravenous therapy using normal saline, dextrose-supplemented saline or lactated Ringer’s solution may have different effects on metabolic balance. These results deserve confirmation in a well-controlled, randomized study. In the meantime, we advise a fluid therapy that takes into account the individual clinical and biochemical variables [14,15]. For mild to moderate gastroenteritis presenting with circulating bicarbonate ≤17.0 mmol/L and glucose ≤3.5 mmol/L, fluid therapy with dextrose-supplemented Ringer solution might be considered instead of normal saline, dextrose-supplemented saline or lactated Ringer.

## Figures and Tables

**Table 1:** Demographics and laboratory data of 134 children 5 months to 15 years of age affected by acute gastroenteritis presenting with mild to moderate dehydration and failure of oral rehydration. Laboratory values are given at baseline and 4–6 h after intravenous fluid therapy at a constant rate of 70 mL/m^2^ body surface area per hour. Data are presented as frequency or as median and interquartile range.

	All Cases	Normal Saline	Dextrose-Supplemented Saline	Lactated Ringer Solution
**N**	134	56	48	30
**Gender (females:males)**	56:78	19:37	29:19 *	8:22
**Age, years**	2.4 (1.2–5.1)	3.6 ^◆^ (1.3–7.7)	2.2 (1.6–4.6)	1.8 (1.0–2.7)
**Whole blood concentration**				
**Ionized sodium, mmol/L**				
At baseline	134 (131–137)	135 (133–137)	133 (130–136)	135 (131–138)
4–6 h later	135 (133–138)	136 (133–138)	135 (133–138)	135 (133–137)
**Ionized potassium, mmol/L**				
At baseline	4.2 (3.8–4.6)	4.3 (3.8–4.9)	4.2 (3.9–4.5)	4.2 (3.5–4.5)
4–6 h later	3.8 (3.4–4.2)	4.0 (3.4–4.4)	3.8 (3.5–4.1)	3.7 (3.2–4.0)
**Ionized chloride, mmol/L**				
At baseline	104 (102–107)	104 (102–107)	103 (100–104)	108 ^✙^ (104–111)
4–6 h later	107 (104–110)	107 (103–109)	107 (105–109)	107 (105–113)
**L-lactate ≥2.5 mmol/L**				
At baseline	0	0	0	0
4–6 h later	0	0	0	0
**Glucose, mmol/L**				
At baseline	4.4 (3.7–5.6)	4.9 (4.3–6.4)	3.6 ^▪^ (3.2–4.3)	4.4 (3.9–6.8)
4–6 h later	4.6 (3.9–5.7)	4.6 (3.8–5.7)	5.3 ^▪^ (4.3–6.6)	4.3 (3.7–4.8)
**Plasma bicarbonate, mmol/L**				
At baseline	17.1 (14.7–20.2)	19.9 (15.9–22.3)	16.1 (14.7–18.6)	15.2 ^✙^ (13.7–17.7)
4–6 h later	19.0 (16.0–21.6)	19.3 (16.0–22.5)	19.1 (16.2–21.3)	18.5 (15.6–20.3)
**Blood, anion gap, mmol/L**				
At baseline	12 (9–16)	10 (8–14)	14 ^☩^ (11–17)	12 (9–15)
4–6 h later	9 (6–12)	9 (7–12)	9 (7–11)	7 (4–12)

* *P* < 0.02 versus normal saline and lactated Ringer; ^◆^ *P* < 0.05 versus normal saline with 5% dextrose and lactated Ringer solution; ^✙^ *P* < 0.05 versus normal saline and dextrose-supplemented normal saline; ^▪^ *P* < 0.05 versus normal saline and lactated Ringer solution; ^☩^ *P* < 0.05 versus normal saline. No missing data.

## References

[1] Moritz M.L., Ayus J.C. (2015). Maintenance Intravenous Fluids in Acutely Ill Patients. N. Engl. J. Med..

[2] Santi M., Lava S.A.G., Camozzi P., Giannini O., Milani G.P., Simonetti G.D., Fossali E., Bianchetti M.G., Faré P.B. (2015). The great fluid debate: Saline or so-called "balanced" salt solutions?. Ital. J. Pediatr..

[3] Semler M.W., Kellum J.A. (2019). Balanced Crystalloid Solutions. Am. J. Respir. Crit. Care Med..

[4] Feld L.G., Neuspiel D.R., Foster B.A., Leu M.G., Garber M.D., Austin K., Basu R.K., Conway E.E., Fehr J.J., Hawkins C. (2018). Clinical Practice Guideline: Maintenance Intravenous Fluids in Children. Pediatrics.

[5] Guarino A., Ashkenazi S., Gendrel M., Vecchio A.L., Shamir R., Szajewska H. (2014). European Society for Pediatric Gastroenterology, Hepatology, and Nutrition/European Society for Pediatric Infectious Diseases Evidence-Based Guidelines for the Management of Acute Gastroenteritis in Children in Europe. J. Pediatr. Gastroenterol. Nutr..

[6] Canziani B.C., Uestuener P., Fossali E., Lava S.A.G., Bianchetti M.G., Agostoni C., Milani G.P. (2017). Clinical Practice: Nausea and vomiting in acute gastroenteritis: Physiopathology and management. Eur. J. Nucl. Med. Mol. Imaging.

[7] Mazzoni M.B., Milani G.P., Bernardi S., Odone L., Rocchi A., D’Angelo E.A., Alberzoni M., Agostoni C., Bianchetti M.G., Fossali E.F. (2019). Hyponatremia in infants with community-acquired infections on hospital admission. PLoS ONE.

[8] Reid S.R., Losek J.D. (2009). Rehydration: Role for early use of intravenous dextrose. Pediatr. Emerg. Care.

[9] Levy J.A., Waltzman M., Monuteaux M.C., Bachur R. (2013). Value of Point-of-care Ketones in Assessing Dehydration and Acidosis in Children with Gastroenteritis. Acad. Emerg. Med..

[10] Severs D., Hoorn E.J., Rookmaaker M.B. (2014). A critical appraisal of intravenous fluids: From the physiological basis to clinical evidence. Nephrol. Dial. Transplant..

[11] Moritz M.L. (2018). Why 0.9% saline is isotonic: Understanding the aqueous phase of plasma and the difference between osmolarity and osmolality. Pediatr. Nephrol..

[12] Burnett R.W., Covington A.K., Fogh-Andersen N., Külpmann W.R., Lewenstam A., Maas A.H., Muller-Plathe O., Sachs C., Siggaard-Andersen O., Vankessel A.L. (2000). Recommendations for Measurement of and Conventions for Reporting Sodium and Potassium by Ion-Selective Electrodes in Undiluted Serum, Plasma or Whole Blood. Clin. Chem. Lab. Med..

[13] Lavagno C., Milani G.P., Uestuener P., Simonetti G.D., Casaulta C., Bianchetti M.G., Fare P.B., Lava S.A.G. (2017). Hyponatremia in children with acute respiratory infections: A reappraisal. Pediatr. Pulmonol..

[14] Kampmeier T.G., Rehberg S., Ertmer C. (2014). Evolution of fluid therapy. Best Pr. Res. Clin. Anaesthesiol..

[15] Sterns R.H., Silver S. (2003). Salt and water: Read the package insert. QJM Int. J. Med..

